# Migratory Movements and Its Effect on the Epidemiology and Clinical Profile of HIV Infection in Quito, Ecuador

**DOI:** 10.1155/arat/6901278

**Published:** 2025-08-25

**Authors:** A. F. Montalvo Vásquez, María José Molestina, Rosa Terán Terán, I. E. Viteri Basso, Daniel Garzón Chávez, Jesús Elías Dawaher

**Affiliations:** ^1^ Pontificia Universidad Católica de Ecuador, Quito, Ecuador; ^2^ Hospital Pablo Arturo Suárez, Pontificia Universidad Católica de Ecuador, Quito, Ecuador; ^3^ Colegio de Ciencias de la Salud, Universidad San Francisco de Quito, Quito, Ecuador, usfq.edu.ec

## Abstract

**Objective:**

Establish the effect that migration has on the epidemiology and clinical profile of human immunodeficiency virus (HIV) infection in a specialized HIV clinic of a hospital in Quito, Ecuador.

**Patients and Methods:**

A cross‐sectional descriptive observational study was carried out, through a survey of 293 people living with HIV (PLWHA) between 2017 and 2019, which included sociodemographic and clinical variables that were taken from the medical records of each participant.

**Results:**

90.4% of PLWHA were men. 74.4% reported a monthly economic income lower than the basic wave (46.8% were unemployed). 51.9% of PLWHA were from Ecuador and 39.9% of Venezuelan nationality. 39.5% of the PLWHA had a late‐advanced diagnosis of the disease. And 78.2% of PLWHA had a current viral load (VL) less than 50 copies/mm^3^. When comparing national and foreign patients, variables with statistically significant differences were found between both groups, and no differences were found in other variables.

**Conclusions:**

In certain aspects, there are no statistical differences between Ecuadorians and Venezuelans such as sex, bisexual sexual preference, marital status, and changes in antiretroviral scheme, among others. It was found that there were higher levels of education, employment rates, and male–female ratio in the foreign population compared to the national population, where probably social dynamics are playing a key factor.

## 1. Background

HIV infection is transmitted by exposure to fluids (mainly blood and semen) through sexual intercourse or vertical transmission; the infection leads to a continuous destruction of CD4+ T lymphocytes [1]. In 2015, The Joint United Nations Program on HIV/acquired immunodeficiency syndrome (AIDS) (UNAIDS) established a goal for the year 2020: the 90‐90‐90 strategy for the HIV pandemic; ensuring that 90% of PLWHA know their serological status, of which 90% are on antiretroviral therapy (ART), and of the latter 90% have a suppressed VL [2].

Worldwide, for the year 2020, it was estimated that there were 37.7 million PLWHA, of which 84.4% knew their diagnosis, 73% had access to ART, and 90% had a suppressed VL [3]. In Ecuador, the same year, there were approximately 45,000 PLWHA, 83% of them knew their diagnosis, 80% had access to ART, and 80% had a suppressed VL [4].

The HIV epidemic is considerably influenced by migration, hence the need to include the migrant population in programs to combat HIV/AIDS as a key population [2]. Some important situations that predispose the migrant population to HIV infection are commercial sex [5] and lack of a stable sexual partner [6].

After the unprecedented crisis in the region, which arose in Venezuela [7], Latin America witnessed an enormous and rapid rate of migratory movements originating from that country [8], with a significant part of the male sex and young people presenting the highest rates of migration, which is also the group most affected by the HIV pandemic [9]. Ecuador, Colombia, and Peru currently host up to 70% of these migrants [10].

Given the increase in resistance to ART, the scheme based on tenofovir, lamivudine, and dolutegravir (DTG) would improve adherence and efficacy [11], as ruled in 2019 by the Quito Process [12]. Migrants represent up to a third of new diagnoses of HIV infection [13], although some already migrate with the infection, others are involved in transmission networks upon arrival to their destination, mainly the man who have sex with men (MSM) population [9].

## 2. Population and Methods

This research was carried out at the Pablo Arturo Suárez Hospital (PASH), a health unit located in Quito, which has a PLWHA Care Unit (PCU), inaugurated on September 29th, 2017. The population consisted of patients over 18 years, treated at the PCU of the PASH. Type of Study: It is a cross‐sectional descriptive observational study.

### 2.1. Selection Criteria

● Inclusion criteria: Patients with confirmed HIV infection, over 18 years, who agree to be part of the study, sign the informed consent, and information was available to be recovered from medical records.
● Exclusion criteria: Patients under 18 years, without diagnostic confirmation and who do not wish to be part of the research before or during the study, were not in capability to provide a written consent, or all the information was not full registered in the medical records.

### 2.2. Data Collection

A survey was carried out to the patients included in the research, and this information was complemented with the data from medical records such as VL and CD4+ level, evaluation in successive consultations, clinical evolution, and sociodemographic. Software used was statistical program SPSS 22.0 Version and excel. Descriptive statistics were calculated for all variables, and for inferential statistics, we used Chi square, with Fisher’s accuracy test and odds ratio (OR) calculate risk, according to the appropriate variable. Also, multivariate regression and a binary logistic regression were done. The objective of the study was to know the effect of migration on the epidemiology and clinical profile of HIV infection in the PCU of a second level hospital of Ecuador.

## 3. Results

A total of 293 patients with confirmed HIV infection were included, 265 men (90.4%) and 28 women (9.6%). 62.5% of PLWHA were between 25 and 39 years, and it was found that 56% of these patients were MSM. 45.4% of PLWHA had a higher level of education. 74.4% of the total sample had a monthly economic income lower than the basic basket and 46.8% were unemployed. 86.3% of PLWHA did not have health insurance (as it is a free hospital from the Health Ministry), and 34.5% reported not having any financial support at all. 51.9% of the patients were Ecuadorian, and 48% were foreigners (mostly Venezuelans who represented 39.9% of the total sample). 89.4% of the patients had an indefinite permanence plan in Ecuador. 14.7% of users reported use of recreational drugs.

**Figure 1:**
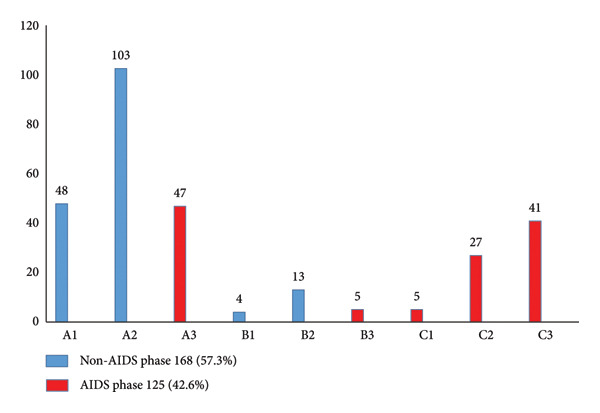
CDC categories of the patients include in the study description of the total number of patients according to the centers for disease control and prevention (CDC), categories, and number of patients that present: acquired immunodeficiency syndrome (AIDS). (A category) asymptomatic, (B category) unspecific manifestations (C category) AIDS—indicator condition, numeration is determinated by lymphocytes T CD4+ count at the diagnosis: (1) higher than 500 cells by mm^3^, (2) between 200 and 500 mm^3^, and (3) less than 200 cells by mm^3^.

81.2% of PLWHA were diagnosed with HIV infection in the 2014–2019 period. 39.5% of PLWHA were diagnosed with a late‐advanced form of the disease, and an additional 42.6% of the patients progressed to the AIDS phase (at the time of diagnosis, due to abandonment of treatment and consultation, or referrals from other units already in the AIDS phase). 87.7% of the patients were taking a first current ART scheme based on two nucleoside reverse transcriptase inhibitors (NRTIs) and one non‐nucleoside reverse transcriptase inhibitor (NNRTI). Most of them never required a change in the ART regimen (81.9% of patients). 78.2% were found to have a VL less than 40 copies/mm^3^ (undetectable) and the majority, 51.5% had a CD4+ T lymphocyte count between 200 and 500 cells/mm^3^ (Figure 1). 49.1% of PLWHA did not have HIV‐related diseases (opportunistic infections, non‐AIDS–defining infections but related to HIV, AIDS‐defining cancer, and other sexually transmitted diseases) (Tables 1 and 2).

**Table 1:** Sociodemographic characteristics and description of all patients included in this study.

Variable	Condition	*N* = 293 (%)
Sex	Men	265 (90.4%)
	Female	28 (9.6%)

Age	18–24 years	43 (44.7%)
	25–39 years	183 (62.5%)
	40–64 years	67 (22.9%)
	> 65 years	0 (0.0%)

Sexual preference	Heterosexual	96 (32.8%)
	Bisexual	33 (11.3%)
	MSM^∗^	164 (56.0%)

Level of instruction	None	1 (0.3%)
	Elementary	20 (6.8%)
	High school	120 (41.0%)
	Third level	133 (45.4%)
	Fourth level	19 (6.5%)

Monthly economic income (basic basket—$716.14)	Less than	218 (74.4%)
	Equal to	58 (19.8%)
	Greater than	17 (5.8%)

Work activity	Yes	156 (53.2%)
	No	137 (46.8%)

Consumption of recreational drugs	Injection drugs	0 (0.0%)
	Inhaled drugs	6 (2.0%)
	Alcohol	14 (4.8%)
	Tobacco	12 (4.1%)
	Tobacco + alcohol	11 (3.8%)
	None	250 (85.3%)

Nationality	Ecuador	152 (51.9%)
	Colombia	16 (5.5%)
	Venezuela	117 (39.9%)
	Perú	3 (1.0%)
	Cuba	3 (1.0%)
	México	1 (0.3%)

Permanence plan in Ecuador	Indefinite	262 (89.4%)
	Temporary	31 (10.6%)
	Passing	0 (0.0%)

Country of diagnosis	Ecuador	222 (75.8%)
	Venezuela	62 (21.2%)
	Colombia	5 (1.7%)
	Alemania	2 (0.7%)
	Cuba	2 (0.7%)

^∗^MSM: Men who have sex with men.

**Table 2:** Clinical features.

Variable	Condition	*N* = 293 (%)
Date of diagnosis	1994–2003	9 (3.1%)
	2004–2013	46 (15.7%)
	2014–2019	238 (81.2%)

Diagnosis stage	Late	38 (13.0%)
	Advanced	114 (38.9%)
	Opportune	55 (18.8%)
	Not available	86 (29.4%)

Current diagnosis	Non AIDS phase	224 (76.5%)
	AIDS phase	69 (23.5%)

Number of ART schemes previously received	One	44 (15.0%)
	Two	8 (2.7%)
	Three	1 (0.3%)
	None	240 (81.9%)

Previous ART scheme	2 NRTIs + NNRTI	31 (10.6%)
	2 NRTIs + INI	2 (0.7%)
	2 NRTIs + PI	20 (6.8%)
	None	240 (81.9%)

Change of ART scheme	Yes	53 (18.1%)
	No	240 (81.9%)

Cause of change of ART scheme	Virologic failure	18 (6.1%)
	Adverse effects	19 (6.5%)
	Drug availability	14 (4.8%)
	Transition to DTG/3TC	2 (0.7%)
	Not applied	240 (81.9%)

Cause of first consultation in PASH	Weight loss	1 (0.3%)
	Fever	23 (7.8%)
	Diarrhea	7 (2.4%)
	Adenopathies	1 (0.3%)
	Continue treatment/transfer	225 (76.8%)
	Opportunistic infections	25 (8.5%)
	Fever + diarrhea + weight loss	11 (3.8%)

Current ART scheme	2 NRTIs + NNRTI	257 (84.0%)
	2 NRTIs + INI	6 (2.0%)
	2 NRTIs + PI	22 (7.5%)
	INI + PI	7 (2.4%)
	INI + NNRTI + PI	1 (1.0%)

Current viral load (copies/mm^3^)	Less than 50	229 (78.2%)
	51–500	40 (13.7%)
	501–1000	3 (1.0%)
	Greater than 1000	21 (7.2%)

Current seric CD4+ T lymphocytes level (cells/mm^3^)	< a 200	31 (10.6%)
	201–499	151 (51.5%)
	> a 500	111 (37.9%)

HIV‐related diseases	STD	66 (22.5%)
	Hepatitis B	8 (2.7%)
	STD + hepatitis B	5 (1.7%)
	Opportunistic diseases	48 (16.3%)
	Opportunistic diseases + STD	15 (5.11%)
	Opportunistic diseases + hepatitis B	3 (1.0%)
	None	148 (50.5%)

*Note:* Descriptive of all patients included. Diagnosis stage, treatment, lab values, and comorbidities are presented. 3TC = emtricitabine.

Abbreviations: AIDS = acquired immunodeficiency syndrome, ART = antiretroviral treatment, DTG = dolutegravir, HIV = human immunodeficiency virus, INI = integrase inhibitor, NRTIs = nucleoside reverse transciptase inhibitors, NNRTI = non‐nucleoside reverse transciptase inhibitor, PASH = Pablo Arturo Suarez hospital, PI = protease inhibitor, and STD = sexually transmitted diseases.

A correlation was made between the variables and nationality. The indefinite permanence plan in Ecuador, sexual preference (MSM) *p* < 0.001 and OR 0.24 (0.61–0.24), and age (25–39 years old) *p* < 0.008 and OR 0.5 (0.32–0.85), and the ART scheme was similar between foreign and national citizens with a *p* < 0.05 and OR 0.96 (0.48–1.93). Regarding the level of education, 56% of the foreign population had third level studies, compared to 35.5% of Ecuadorian PLWHA who had high school studies with a *p* value < 0.001 and OR 2.1 (1.3–3.4). 35.4% of foreign participants and 57.2% of nationals were unemployed with a *p* value = 0.012 and OR 2.4 (1.5–3.9). 33.3% of foreign PLWHA were in the AIDS phase of the disease compared to 51.3% of national PLWHA, with a *p* value = 0.035 and OR 2.11 (1.3–3.4). 49.6% of foreigners had already been diagnosed of HIV infection before migrating to Ecuador (Table 3). 90.7% of foreigners had lived in Ecuador for more than a year, and 78.0% of foreigners had an indefinite permanence plan in Ecuador. 75.2% of foreign PLWHA (*n* = 103) did not receive any ART prior their arrival to Ecuador.

**Table 3:** Sociodemographic and clinical characteristics associated with nationality.

Variable	Condition	*N* = 152 Ecuadorian (%)	*N* = 141 Foreigners (%)	*X* ^2^ test *p* value	OR (95% CI)
Age	18–24 years old	33 (21.7%)	10 (7.0%)	0.003	3.6 (1.7–7.69)
	25–39 years old	84 (55.2%)	99 (70.2%)	0.008	0.5 (0.32–0.85)
	40–64 years old	35 (23.0%)	32 (22.6%)	0.9462	1.02 (0.6–1.76)

Sex	Men	132 (86.8%)	133 (94.3%)	0.191	2.5 (1.07–5.9)
	Women	20 (13.1%)	8 (5.6%)		

Marital status	Single	109 (71.7%)	109 (77.3%)	< 0.001	0.7 (0.42–1.23)
	Married	20 (13.1%)	10 (7.0%)		1.98 (0.89–4.4)
	Divorced	5 (3.2%)	5 (3.5%)		0.96 (0.26–3.27)
	Free union	18 (11.8%)	16 (11.3%)		1.04 (0.5–2.13)
	Widower	0 (0.0%)	1 (0.7%)		^∗^

Sexual preference	Heterosexual	65 (42.7%)	31 (21.9%)	0.001	2.7 (1.6–4.4)
	Bisexual	19 (12.5%)	14 (9.9%)		1.3 (0.6–2.7)
	MSM	68 (44.7%)	96 (68.0%)		0.24 (0.61–0.24)

Level of instruction	None	1 (0.6%)	0 (0.0%)	< 0.001	^∗^
	Elementary	18 (11.8%)	2 (1.4%)		9.4 (2.14–41.33)
	High school	75 (49.3%)	45 (31.9%)		2.1 (1.3–3.4)
	Third level	54 (35.5%)	79 (56.0%)		0.36 (0.22–0.58‐)
	Fourth level	4 (2.6%)	15 (10.6%)		4.4 (1.4–13.6)

Permanence plan in Ecuador	Indefinite	152 (100.0%)	110 (78.0%)	< 0.001	^∗^
	Temporary	0 (0.0%)	31 (21.9%)		

Financial supporting	Relatives	115 (75.6%)	77 (54.6%)	< 0.001	0.39 (0.24–0.64)
	No one	37 (24.3%)	64 (45.3%)		

Employment	Yes	65 (42.7%)	91 (64.5%)	0.012	2.4 (1.5–3.9)
	Not	87 (57.2%)	50 (35.4%)		

Residence	Own house	66 (43.4%)	1 (0.7%)	< 0.001	0.009 (0.001–0.07)
	Renting	85 (55.9%)	140 (99.2%)		
	Temporary shelter	1 (0.6%)	0		

Current diagnosis	AIDS phase	78 (51.3%)	47 (33.3%)	0.035	2.11 (1.3–3.4)
	Non AIDS phase	74 (48.6%)	94 (66.6%)		

Current ART scheme	2 NRTIs + NNRTI	133 (87.5%)	124 (87.9%)	< 0.001	0.96 (0.48–1.93)
	2 NRTIs + INI	2 (1.3%)	4 (2.8%)		1.04 (0.52–2.1)
	2 NRTIs + PI	11 (7.2%)	11 (7.8%)		
	INI + PI	5 (3.2%)	2 (1.4%)		
	INI + NNRTI + PI	1 (0.6%)	0 (0.0%)		

Country of diagnosis	Ecuador	151 (99.3%)	71 (50.3%)	< 0.001	0.01 (0–0.05)
	Foreign	1 (0.6%)	70 (49.6%)		

Abbreviations: AIDS = acquired immunodeficiency syndrome, ART = antiretroviral treatment, HIV = human immunodeficiency virus, INI = integrase inhibitor, MSM = men who have sex with men, NRTIs = Nucleoside reverse transcriptase inhibitors, NNRTI = Non‐nucleoside reverse transcriptase inhibitor, and PI = protease inhibitor.

^∗^One category equal to zero, not possible to calculate.

To control potential cofounders, a multivariate regression was done, analyzing the condition of Ecuadorian or foreign and HIV infection duration, ART change schema, coinfections, and drug consumption, and the model was adjusted with statistical significance (Chi^2^ test 63.82, d*f* 23, *p* = 0.001). HIV infection duration presented association with a coefficient *b* = 0.85, *p* = 0.03 OR = 2.34, and ART change schema *b* = 3.13, *p* = 0.018 OR = 23.07, therefore must be considered as potential confounders.

In a binary logistic regression, we evaluated AIDS progression (VIH presence vs. AIDS) and the variables presented in Table 3, and the model was adjusted with statistical significance (Chi^2^ test 50.752, d*f* 23 *p* = 0.002). Two variables presented association: sexual orientation (bisexual) with a coefficient *b* = 1.276, *p* = 0.001 and a OR = 3.58 and financial supporting (not financial support) with a coefficient *b* = 1.574, *p* = 0.002, and a OR = 4.85. Employment status did not present a significant association with AIDS progression.

## 4. Discussion

Of the 293 patients, more than 90% of them were men. There were a male: female ratio of 9.4:1 (6:1 in Ecuadorian patients and 16.6:1 in the foreign population) (Table 1), a result that is far from the worldwide epidemiology, which shows a frank feminization of the epidemic with a male: female ratio of 1.12:1 [3] and even in the Ecuadorian epidemiology that reports a male: female ratio in 2:1 [4]. This is likely explained by the large number of migrants (most of them are men) and MSM treated at PASH PCU, a population group where the HIV/AIDS epidemic is concentrated [14].

More than 60% of PLWHA users belonged to the 25–39 years old age range, showing a trend of the disease for the economically and sexually active population. Similar data are reported in Latin America [15] and in Ecuador [4, 16]. In the comparative analysis, there was a significant statistical difference between nationalities, where 55.2% of national citizens presented low risk in comparison with international citizens (70.2%) that fell within the age range of 25–39 years. These findings align with the information reported by the National Institute of Statistics and Census (NISC), which indicates that foreign citizens aged 18 to 29 years are the most frequent entrants and departures from Ecuador. Additionally, Venezuelans aged 18 to 42 years are identified as the predominant nationality entering Ecuador, Colombia, and Peru [17].

Around half of the patients included in the study were foreigners, out of which majority of the total participants were Venezuelan PLWHA, these data are confirmed by NISC that show that the migrant population from Venezuela has, by far, the largest flows into and out of Ecuador [18]. Vast majority of PLWHA indicates that they had an indefinite permanence plan in Ecuador. National citizens did not have among their plans to leave Ecuador, and it is inferred, based on the literature, that foreign citizens prefer to stay in our country, due to the reception policies and the equitable treatment toward them [19], as well as the statistics show that since 2015, the Venezuelan population has opted for an indefinite residence in Ecuador [20].

There is a clear significant statistical difference (*p* < 0.001) between sexual preference groups; majority of the foreigners participants reported that they have sexual preference as MSM (in the group of Ecuadorian nationality this sexual preference represented 44.7% of the sample and a low risk), and high risk in bisexual participants with 3.5 times higher (Table 3), similar to the national data, which indicate that the HIV epidemic is concentrated in key population groups, such as MSM, and transfeminine women (TFW) mainly in Quito and Guayaquil [4, 14]. This is also consistent with what is described in other countries such as Venezuela [21] and Colombia [22]. In Latin America, it has been reported that male sex is the one that has shown the highest rates of migration in the region and on the other hand, the MSM population is the one that represents the highest transmissibility of HIV infection [9]. Therefore, the high migratory flow in the country, which is also predominantly male, influences the epidemiological situation of HIV in Ecuador and would explain the differences between PLWHA of Ecuadorian and Venezuelan origin in this study. This impact has been reported in the literature in neighboring countries that face the same problem [23].

Regarding other demographic factors, almost half of PLWHA indicated that they had a higher level of education (Table 1), which goes against the statement that says, “if children and adolescents receive adequate educational instruction, this would improve their preventive knowledge against HIV infection” [24], and present a clear difference (*p* < 0.001) with the Ecuadorian group in which one‐third had higher level education and around three times less low risk. Probably, this may suggest that foreigners with a higher level of education migrate in search of better opportunities and optimal control of their disease.

Most of the participants had a monthly economic income lower than the basic wave, and around half of them reported being unemployed. This can be partly explained by the economic crisis and by the fact that PLWHA may fear discriminatory attitudes from the general population if their serological status becomes known, which could also cause this population to self‐limit in their work development [25]. However, the foreign PLWHA had a higher employment rate (*p* = 0.012) around two times more risk than the nationals. This is relevant because the literature shows that lack of employment is one of the key factors that drive migration [26]. Also, patients who did not receive any financial support presented 4.8 times higher risk to AIDS progression. The world literature reports that drug use, whichever it is, is associated with a higher risk of acquiring HIV infection and loss of follow‐up of PLWHA, nevertheless in this study, four‐fifths of participants reported not consuming any type of recreational drug [27].

Most (81.2%) of PLWHA present in this study were diagnosed during the period between 2014 and 2019. This statistic reflects both the rising number of cases and the widespread utilization of diagnostic tests, along with the reinforcement of screening campaigns, as was reported by the health ministry department [4].

Almost two‐fifths of participants had a late‐advanced diagnosis of the disease, a fact that was limited in part because in around a third of total participants, we did not have access to the initial CD4+ T lymphocyte count (because they were referred to from other health centers, and databases were not available), and we include these patients in the study with CD4+ T lymphocyte count values obtained in the first contact that we have with them.

Throughout 2020, it was observed that one 18.4% of new diagnoses in Ecuador were associated with a CD4+ count less than 200 cells/mm^3^ [4, 28]. By comparison, Latin America witnessed 29% of new HIV infections in 2019 presenting a similar low CD4+ counts level [18]. Analysis of accumulated data shows that 42.6% of participants progressed to the AIDS phase of disease at some point (Table 3). Although national data characterizing the cumulative percentage of patients with AIDS are not registered or are not available; findings from a 2020 observational retrospective study conducted in Guayaquil identified that 85.3% of participants were in the AIDS category of the disease [29], a markedly higher proportion than this study revealed.

Regarding to viral load, 78.2% of PLWHA included in this study accomplish with undetectable criteria (less than 40 copies/mm^3^), nevertheless an appropriate comparison with national data from 2020 become imprecise because official records reported 80% PLWHA in ART as suppressed VL (less than 1000 copies/mm^3^) as a goal established by the World Health Organization (WHO), but it does not discriminate those with undetectable VL (which is assumed to be a significantly lower percentage). Showing a remarkable goal to improve adequate records, since the primary objective of treatment in the PCU is to achieve undetectable viral loads in patients [4].

CD4+ count follow‐up was performed to categorize the patients according to the Center for Disease Control and Prevention (CDC) classification (where the stages are progressive and not regressive), showing that the majority (103 users, 35.1%) were in the A2 stage. Also, it was identified that one in 10 patients, who did not accomplish the secondary objective of treatment, had a count below 200 cells/mm^3^.

It is important to be aware that results in clinical patients’ evolution were done with treatments currently used in 2017. DTG was only became available in Ecuador in mid‐2021 [1] through the National HIV Strategy, this drive a massive rotation in 87% of participants that for its initial scheme received an ART scheme based on 2 NRTIs + 1 NNRTI which was established in the PLWHA Clinical Practice Guideline of WHO 2003 [29].

In conclusion, it is evident that although in certain significant aspects, there is no statistical difference between Ecuadorian and Venezuelan population such as age range between 40 and 64 years old, sex distribution, sexual preference: bisexual, and marital status. While aspects that present a difference includes Ecuador, permanence plan: indefinite in Ecuador, refusal of ART prior to the first medical consultation at the study center, and not having had changes in ART (they maintain the initial regimen, and the nationality is not an associated with high risk), regarding to country of diagnosis: around the half of foreign were diagnosed in their original country.

Among other aspects, there were differences in the PLWHA when their nationality was evaluated: higher level of education and higher employment rates in the foreign population compared to the national population. Regarding the progression of the disease, the majority of national PLWHA were in the AIDS phase of the disease compared to the foreign population (around two times higher risk that foreign); however, after multivariate regression confounders such as HIV infection duration and ART change schema make an uncertain clear difference between Ecuadorians and foreign population, and it cannot be asseverated that the two groups are distinct. Probably social dynamics also play a key factor for differences in the two groups [30–32].

Migration modifies social demographic profile of a country, in the context of HIV introducing a different patient profile with their own clinical and social variables. A workflow for health centers could include international communication between health centers, promoting social support networks, working in cultural acceptance of health services, capacitation in cultural awareness given that to understand the patients’ different characteristics between national and foreign patients is crucial for establishing specific public health measures between groups.

## 5. Limitations

This study was carried out in only one center and has a transversal design. The result cannot be extrapolated to all migrant situations in Ecuador; also variables cannot be evaluated through time and migration variables are highly changing. Identification of confounders such as HIV infection duration and ART change schema must be considered through explaining the difference to the Ecuadorian and foreign groups. Also, for all analysis in this study, the first available CD4+ T lymphocyte count was used, due to problems in accessing records or losing them during migration events.

## Ethics Statement

Approval was obtained from the ethics committee of Pontificia Universidad Católica del Ecuador. The procedures used in this study adhere to the tenets of the Declaration of Helsinki. CEISH: SB‐CEISH‐POS‐357.

## Consent

Informed consent was recollected from all of the study participants.

## Conflicts of Interest

The authors declare no conflicts of interest.

## Funding

No funding was received for conducting this study.

## Data Availability

The data that support the findings of this study are available from the corresponding author upon reasonable request.
