# Correction to “Migratory Movements and Its Effect on the Epidemiology and Clinical Profile of HIV Infection in Quito, Ecuador”

**DOI:** 10.1155/arat/9790420

**Published:** 2026-07-26

A. F. Montalvo Vásquez, M. J. Molestina, R. T. Terán, I. E. Viteri Basso, D. G. Chávez, and J. E. Dawaher, “Migratory Movements and Its Effect on the Epidemiology and Clinical Profile of HIV Infection in Quito, Ecuador,” *AIDS Research and Treatment*, 2025, no. 1 (2025): 1–8, https://doi.org/10.1155/arat/6901278.

The article was originally published incorrectly as a Review Article. The correct article type is Research Article, and the article has been updated to reflect this.

Additionally, the first author’s name was incorrectly stated as Andrés Montalvo Vásquez.

The author’s correct name is Andrés Fernando Montalvo Vásquez and has been corrected in the article.

We apologize for these errors.

